# Exploring the Impact of Music Education on the Psychological and Academic Outcomes of Students: Mediating Role of Self-Efficacy and Self-Esteem

**DOI:** 10.3389/fpsyg.2022.841204

**Published:** 2022-02-08

**Authors:** Jian Sun

**Affiliations:** School of Music and Dance, Xihua University, Chengdu, China

**Keywords:** psychological wellbeing, self-cognition theory, self-determination theory, music education, self-esteem, self-efficacy, pandemic

## Abstract

In recent years, there has been a growing interest in scholars and practitioners to explore the factors that lead to an improvement in Students’ psychological wellbeing. Due to the tough challenges faced by students during their academic life, severe issues of stress, anxiety, and other mental health issues emerge, which affect their academic performance and have a long-lasting impact on their future careers. The pandemic accelerates the stress levels, anxiety, and mental issues of students. The main purpose of this study was to explore how music education impacts on Students’ psychological wellbeing and academic performance. This study also investigates the mediating effect of self-esteem and self-efficacy. To the best of our knowledge, there has been little to no study exploring the relationship of music education on the psychological wellbeing and performance of students, especially from the perspective of Asian countries. This study was conducted in undergraduate and graduate institutions of China. This study was quantitative in nature and data were collected from 319 respondents. The structural equation modeling (SEM) technique was employed for data analysis. Results reveal that music education has a significant positive impact on psychological wellbeing, which improves Students’ academic performance. Moreover, psychological wellbeing also has a significant and positive impact on Students’ academic performance. Self-efficacy and self-esteem significantly mediate the relationship between music education and psychological wellbeing. The findings of this study open new avenues for future research in music education and psychological wellbeing. This study suggests that the policymakers and practitioners should make such policies that encourage educational institutes to adopt music education to improve the psychological wellbeing of students.

## Introduction

A huge amount of scientific research shows that the pandemic and its associated illness has a significant influence on the behavoir and mental health of individuals (Loades et al., 2020; Luo et al., 2020), with only a few studies indicating contrary (Gijzen et al., 2020). Even during the month of April 2020, when most people were on lockdown due to the pandemic, mental health hotlines in the United States saw 100-fold increases. Many healthcare centers have reported more casualties from suicide, likely due to severe psychological problems, than from the novel coronavirus. Several persons who in the past were not into wellbeing, now have more chance of getting into trouble during the pandemic regarding their health concerns due to the inability of generating more economic means. The pandemic has an influence on a wide range of people, but because of the uncertainty surrounding academic progress, social life, and employment, college students are among the most severely impacted (Kim et al., 2020). Students all over the world were experiencing rising feelings of anxiety, negative moods, low self-esteem, psychological symptoms, drug addiction, and suicidal behaviors even before the epidemic (Huo et al., 2020; Nawaz et al., 2020a; Wu et al., 2020).

Consequently, students now require more resources and help to deal with the health-related adversities due to COVID-19. Students would be well-treated during this pandemic if the officials associated with the health of university students have pre-defined measures for coping with the negative impacts of the pandemic on psychological factors. These consequences provide enough insight about appropriate and necessary measures to be taken for addressing the health issues of students (Hunt and Eisenberg, 2010). College students have traditionally underused psychiatric and counseling services. Recognizing which sub-populations may be affected by certain mixtures of psychological effects can help with specific therapies, effective treatment, and coping methods for those who are most at risk. To combat the possibility of illness spreading, the government has taken a number of steps. Isolation and loneliness, travel limitations, gathering restrictions, travel quarantine, trading closures, working from home, self-isolation, lockdowns, curfews, and university closures are all examples of these methods (Hao et al., 2020; Nawaz et al., 2020b; Sattar et al., 2020). Governments in a range of countries have imposed a lockdown or curfew as a precaution against the rapid spread of the virus (Abdullah et al., 2020; Paital et al., 2020; Boonroungrut et al., 2022). Such policies have an adverse impact on business, schooling, healthcare, and entertainment across the globe.

Many institutions worldwide have delayed or canceled all campus events to reduce crowding and thereby viral spread. On the other hand, these policies have greater economic, medical, and social consequences for both postgraduate and undergrad communities. Owing to the cessation of classroom training at several colleges and institutions, undergraduate and graduate students can now benefit from online instruction (Iyer et al., 2020; Sahu, 2020; Yamin, 2020). This kind of instruction offers an option to minimize either student-to-student interaction or student-to-lecturer contact. Unfortunately, many students are unable to access online education owing to a lack of resources or equipment as a result of the economic and digital gap. COVID-19 has been linked to educational studies in a few papers (Kanneganti et al., 2020; Mian and Khan, 2020; Sandhu and de Wolf, 2020). Graduate practitioners, dental medical students, and radiological trainees are all affected by COVID-19 (Alvin et al., 2020).

This study aims to establish a strong relationship between music education and analyzing the impact of music education on Students’ wellbeing and academic performance in universities. The primary goal of good education is to develop social thinkers who think creatively. While stimulating learners’ vision and imagination, music education may enhance Students’ passions, sentiments, and other non-intellectual variables, fulfilling the goal of nurturing Students’ inventive identities. As a result, music instruction at colleges and universities is an effective way to help students overcome “poorly functioning” personalities (Arora and Singh, 2020). Students may develop their innovative identities and feel a feeling of self and self-efficacy *via* music instruction, allowing them to transcend personality flaws caused by the many negative elements in today’s cultural milieu. When it comes to efforts to improve music instruction in universities and colleges (Chen et al., 2019a).

Researchers believe that education about music at educational institutes could develop creative thinking in students. Such education could be initiated by theorizing the impact of music on regulating the function of psychology for the wellbeing of the students. This could be combined with actual teaching methods to identify a way forward for the improvement of the mental health of students through mixed-method teaching. Students at the post-matriculation level could be advised to actively participate in such music education activities to develop a mastery of music. This would aid in developing open-mindedness in students of this level and inter-communication skills regarding learning would also be improved. It will develop a culture of understanding others at a social level and their sense of self-control would also improve. It would also help them in reducing anxiety and lead to psychological wellbeing, ultimately leading to sound health (Ling et al., 2020).

Generally, it is assumed that teaching music is not only a way of learning an art, but also has a significant role in psychological regulation and treatment; thus, education in music will lead to a specific role in treating the disease at educational institutes because the most important goal is to cultivate students independent personalities. Some music instructors frequently utilize their prowess to intimidate pupils and swamp them with information. Contextual motives now account for a considerable portion of learning outcomes (Bagozzi and Yi, 1988; Hair et al., 2014). The students at this level would consider this challenging for keeping a pace in learning in this discipline if outside motives are removed. Colleges and universities must begin with Students’ actual mental wellbeing and provide colorful music teaching activities, which have been extensively used in the practice of college Students’ mental health work, and the role of psychological regulation function (Zupan and Gadpaille, 2020). It can specifically assist children in improving their psychological health by establishing music education rooms using teaching tools at school level.

This may assist students in realizing the importance of the positive mental ability to develop learning skills by offering appropriate music psychological optional courses centered on topics that are relevant to their learning and lives. It can assist students in forming positive circles of friends and enhancing emotional interactions amongst them (Le Prell et al., 2018). Furthermore, we may use Internet resources to undertake digital psychological counseling exercises, assist students by recommending additional attractive and motivational music compositions, and teach a certain basic understanding of music education, such that learners enjoy getting psychological enjoyment from melody. Simultaneously, the institute’s psychological aspects, utilizing music as a carrier, strengthening communication between students through games, performances, and other means, establishing a decent vibe of assisting individuals, cooperation, and love, in such a delicate way to monitor Students’ self-conscious study stress, help each other avoid depression, and promote a healthy psychological condition (Chen et al., 2019b; Wang et al., 2019). The impact of music education on the wellbeing, psychological, and academic outcomes of the students could be mediated by the well-known concepts of self-esteem and self-efficacy. Self-efficacy is an the confidence of an individual in managing their environment, which determines how they act, perceive, and think about coming occurrences. Self-esteem is a person’s overall positive or negative assessment of their own value. Self-esteem has been linked to happiness, fulfillment, good stress management, and coping with difficult situations (Bandura, 1977; Yıldırım et al., 2017). In the context of our study, both self-esteem and self-efficacy could yield significant results in terms of helping the model and mediating the relationship of music education with Students’ wellbeing and the academic outcomes in the era of the pandemic. Our study mainly focuses on the above-developed relationships in Students’ performance and their wellbeing. This study was based on certain objectives as follows: (1) To assess the relationship of musical education with the psychological wellbeing of the students and academic performance. (2) To evaluate self-esteem as a mediator between wellbeing and the academic performance of students. (3) To evaluate the mediating role of Students’ self-efficacy between music education and psychological wellbeing and academic outcomes during the pandemic.

The study has been structured thus: the first section explains the introduction and supporting literature, while the second section supports the hypothesis development and study model. Research methodology and data analysis have been written up in the third section. The fourth section contains the discussion and concluding remarks.

## Review of Literature and Hypotheses Development

This research study revolves around the impact of music education on the psychological wellbeing of students along with their academic outcomes. Self-esteem and self-efficacy play a mediating role in the relationship of these. These are supported by the following theories.

### Social Cognitive Theory

This theory helps in describing the functionality of humans with an emphasis on processes of an interactive nature. The cognitive activities are assigned a special role by the theory through which individuals could obtain a handful of insight from their surroundings. Individuals could give a reflection of the theory along with mixing of own behaviors and the ideas. This could also regulate the processes of own self-efficacy. The objective behind connecting this theory with music education was to investigate the significance of developments for developing acceptable learning and teaching practices for advanced students. It is critical to have a good theoretical foundation for understanding how learning happens when planning curricula and instructional services for children with outstanding academic ability. To explain human functioning, social cognitive theory stresses a dynamic interactive process between environmental, behavioral, and personal components. This understanding of human connections and functioning became characterized as a set of triadic reciprocal causation (Avotra et al., 2021a). The theory assigns a major role to cognitive processes in which a person may watch others and the environment, reflect on it in conjunction with their own ideas and behaviors, and adjust their own self-regulatory functions as a result. When looking at learning interventions for that demographic, a learning model that stresses the primary role of cognition appears reasonable. Human agency and perceived self-efficacy are components of the social cognitive paradigm that influences cognitive growth and performance. So, a link could be developed in light of this theory toward the role of music education in developing certain cognitive factors in students for psychological wellbeing.

### Self-Esteem Theory

Self-esteem is still one of the most widely studied topics in social psychology. Self-esteem is often thought of as a component of one’s self-concept (Harder, 1994), although it is one of the most significant aspects of one’s self-concept for certain people. Indeed, self-esteem looked to be interchangeable with self-concept in the literature on the self for a time. The link of strong self-esteem with a range of favorable outcomes for individuals and communities as a whole has prompted this attention on self-esteem. Furthermore, there is a general view that boosting one’s self-esteem (particularly that of a child or teenager) is advantageous to both the individual and society (Cast and Burke, 2002). Self-esteem can relate to a person’s total self or specific components of their self, such as how they feel about their social status, ethnic or racial group, physical characteristics, physical prowess, and work or school achievement. Theorists have indeed classified various kinds of self-esteem as contingent vs. non-contingent; visible vs. tacit; genuine vs. fake; steady vs. volatile; worldwide vs. sector-specific. In terms of the complexity of self-esteem, many writers see it as a single, worldwide characteristic, while some others see it as a heterogeneous feature with distinct constituent parts such as the interpersonal, cognitive, and actual self. Differentiation has been made between a false sense of self-worth and a genuine sense of self-worth. Self-esteem which is dependent on meeting certain criteria of achievement or staying true to certain relational or psychological aspirations is referred to as contingent self-esteem (Deci and Ryan, 1995). This is a form of self-aggrandizement related to being ego-involved in certain objectives and obtaining them diligently. This is frequently related to social comparison and is typically associated with narcissism. From the other part, true self-esteem is much more consistent and is founded on a stable and resilient sense of self. Their value would be represented in action, proactive behavior, and vibrancy as an integral component of their self. When it comes to assessing self-esteem, many writers differentiate among explicit and implicit self-esteem, although that is the reflectively unrecognized influence of self-attitude on the judgment of nature vs. self-dissociated objects. In this connection of the theory, self-esteem was identified as a mediator between the relationship of music education and academic outcomes.

### Self-Determination Theory

Self-determination is a key concept in psychology that relates to a person’s ability to make decisions and govern their own lives. This skill is crucial to one’s mental stability and wellbeing. Individuals who have self-determination believe they have self-control in life. This also affects motivation since individuals are much more driven to act if they believe their actions will have an impact on the result. Self-determination was used in a variety of fields, notably education and health care. According to research, having a high level of self-determination can help one succeed in a variety of areas. According to this theory, people can become self-determined when their demands for competency, connectedness, and independence are met. The concept of self-determination emerged from the research of scientists (Deci and Ryan, 1995), who published their views in the book “Self-Determination and Intrinsic Motivation in Human Behavior.”

They established a motivational theory that argued that individuals are motivated by a desire to learn and improve. Self-determination theory is a meta-theory of motivation and personal development, including psychological wellbeing, that is scientifically grounded (Ryan and Deci, 2000). According to the idea, all humans are born with a strong sense of curiosity and a desire to learn, and that certain contextual conditions may either promote or inhibit a person’s feeling of wellbeing, self-regulation, and intrinsic drive to learn. Belonging, competence, and autonomy are three intrinsic and basic psychological demands identified by the theory. Based on this theory, a study related to music participation was conducted by Krause et al. (2019). All these theories provided a strong ground for the mediators used in this study for the Students’ psychological wellbeing and academic outcomes.

### Music Education Relationship With Psychological Wellbeing, Self-Esteem, and Self-Efficacy

A few studies have been conducted in the past to look into the impact of music education on Students’ psychological wellbeing from different perspectives (e.g., Croom, 2014; Demirbatır, 2015; Erginsoy Osmanoğlu and Yilmaz, 2019; Krause et al., 2019; Mehraban, 2020). Through stimulating Students’ imagination and association, music education may enhance Students’ interests, emotions, and other non-intellectual variables, fulfilling the goal of nurturing Students’ inventive personalities. As a result, music instruction at colleges and universities is an effective way to help students overcome their “dysfunctional” personalities. Students may regulate their own identities for developing a feeling of self-efficacy *via* music instruction, allowing them to overcome the numerous personality flaws caused by the many negative forces in today’s cultural context (Arora and Singh, 2020). The significance of music education for treating illness could be effectively performed in educational institutes since it is understood that educating music is a way of tutoring art and has significance in psychological regulation and treatment. Music education’s personal development benefits have gotten less emphasis. Yet, other research continues to link music training to the development of psychological advantages, such as self-efficacy.

Another study of middle school and high school band, choruses, and orchestral Students’ self-efficacy was undertaken by several researchers. The findings revealed a small positive association between musical ability and self-efficacy. Self-efficacy is higher among students who have a higher level of musical talent. While this research shows a link between music ability and self-efficacy, it does not examine the influence of tutoring music on children lacking this ability by contrast (Zelenak, 2015). The benefits of individual music instruction on pupils’ self-esteem have been proven in studies. Despite the fact that all students have identical motor skills, musical ability, and cognitive abilities, there is concrete evidence that children receiving paid piano lessons have a considerable advantage in developing self-esteem compared to counterparts unable to get tutoring on music education (Costa-Giomi, 2004). Researchers back up this assertion by stating that an active passion for music is statistically significant in predicting self-esteem in children who have paid music tutoring (Wu and Lu, 2021). Research into the influence of school-based music programs on personal development has also been undertaken.

The study focused on a music program that offered general music sessions to primary school children during the school day. The control group received no musical education and suffered a drop in self-esteem. The ones who were enrolled for music learning did not experience a drop in self-esteem. The hand drumming music program in Australia was shown to result in self-esteem being boosted by a substantial amount along with reducing problems associated with behaviors in the children who recently completed their training (Faulkner et al., 2010; Rickard et al., 2013). When researchers looked at high school students reporting specific personal reasons for participating in music-related co-curricular activities, they discovered that they cited sentiments of good self-esteem and self-efficacy. Furthermore, researchers discovered that drumming participants in Africa thought of themselves as serving the cause of music. They felt like it was a great thing to be a part of and it boosted their ability in having the satisfaction of self-efficacy in concluding the lengthy research on learners (Barbre, 2013). All these supportive papers suggested relationships between music education and the psychological wellbeing of students, along with the self-esteem and self-efficacy of the students, so we propose the following:

***H_1_.*** *There is a relationship between Music education and psychological wellbeing.*

***H_2_.*** *There is a relationship between Music education and self-esteem.*

***H_3_.*** *There is a relationship between Music education and self-efficacy.*

### Role of Self-Esteem on the Wellbeing of Students Psychologically

According to the research on self-esteem, there is a substantial relationship between self-esteem and psychological wellbeing. However, this relationship differs depending on the sort of self-esteem being studied. Self-esteem, for example, has been shown to play a beneficial influence in boosting psychological wellbeing in a broad body of research. Furthermore, culture has indeed been found to influence the causal relations between self-esteem and happiness. In individualistic civilizations, self-esteem was shown to be more strongly linked to life satisfaction than in collectivist societies. Individuals from individualistic cultures may value their distinctive qualities and personal characteristics, making self-esteem a more important factor. Individuals in collectivist societies, on the other hand, may place a higher emphasis on relational and communal elements of the self. In collectivist societies it is critical to understand which types of self-esteem are favorable to psychological wellbeing (Diener and Diener, 2009; Sowislo and Orth, 2013).

Some researchers studied the relationship between self-esteem and psychological wellbeing by expanding self-esteem studies beyond the individual to the social level. As per the social identity theory, the collective is an important element of the self, and hence assessing the collective self may help people feel better about themselves. Indeed, they discovered that self-esteem was significantly linked with wellbeing in many white, Black, and Asian students in the United States. Even so, once individual self was taken into account, the relationship between collective self-esteem and wellbeing became non-significant for white students, small for Black students, and moderate too strong for Asian students. This shows that culture may have a significant impact on the importance of various sorts of self-esteem (Crocker et al., 1994). Many studies, such as Singhal and Prakash (2021), indicated a significant relationship among the wellbeing of students and self-esteem. Numerous of studies also pointed out the mediating role of self-esteem from different perspectives and found a significant contribution of self-esteem as a mediator (Hesari and Hejazi, 2011; Bajaj et al., 2016). These studies suggested the role of self-esteem as mediator in music education and psychological wellbeing in the context of our study, so we developed the following hypotheses.

***H_4_.*** *There is a relationship between self-esteem and psychological wellbeing.*

***H_7_.*** *Self-esteem mediates between music education and psychological wellbeing.*

### Role of Self-Efficacy and Psychological Wellbeing

People who have a high level of self-efficacy have a can-do attitude, which helps them to perceive obstacles as issues to solve problems rather than avoid them. They also create appropriately challenging objectives for themselves and stick to them with tenacity. Because they are extremely engaged, people with high self–efficacy love life. When they are confronted with difficult events, their confidence in their capacity to control the situation to their advantage leads them to be self-assured. Greater wellbeing, stress control, greater self-esteem, improved physical state, and better illness adaptation and survival are linked to high self-efficacy. On the other hand, poor self-efficacy seems linked to increased symptoms of anxiety and depression, along with decreased rates of psychological wellbeing (Bandura et al., 2003). A lot of studies such as Siddiqui (2015) indicated a significant positive correlation between self-efficacy and the psychological wellbeing and suggested analyzing the relationship in our context of the study. Several researchers concluded the mediation of self-efficacy in various situations in which self-efficacy plays a mediating role between different variables and contexts (Zhao et al., 2005; Molero et al., 2018; Sabouripour et al., 2021). These studies found a positive strong mediation of self-efficacy and helped us in developing the following hypotheses of this study:

***H_5_.*** *There is a relationship between self-efficacy and psychological wellbeing.*

***H_6_.*** *Self-efficacy mediates between music education and psychological wellbeing.*

### Relationship Between Psychological Wellbeing and the Academic Performance

This interesting connection between academic outcomes and Students’ psychological wellbeing had been studied many times in the past and found significant results (Bhat and Siddiqui, 2015; Alkhatib, 2020; Amholt et al., 2020; Gökalp, 2020; Chaudhry and Ikram, 2021). Psychological suffering has been identified as a serious and pressing concern among university students across the world. According to a study done in the United States, psychology is responsible for five of the top six health-related issues. High psychological distress and low psychological wellbeing are two classifications that may be used to describe university students who are suffering from a high level of mental illness. According to a study conducted by experts, university students in Australia discovered that high levels of psychological wellbeing were associated with reduced levels of depression. However, a lack of psychological wellbeing leads to an increase in despair. A scale to evaluate psychological wellbeing and psychological discomfort was used to evaluate the wellbeing of students. The link between discomfort and psychological wellbeing is presented in the research (Bhullar et al., 2014; Roslan et al., 2017; Sharp and Theiler, 2018). All this supporting literature hinted about the connection of the wellbeing of students and academic achievement in terms of performance, so we propose the following hypothesis in this regard:

***H_8_.*** *There is a relationship between psychological wellbeing and Students’ academic performance.*

**FIGURE 1:**
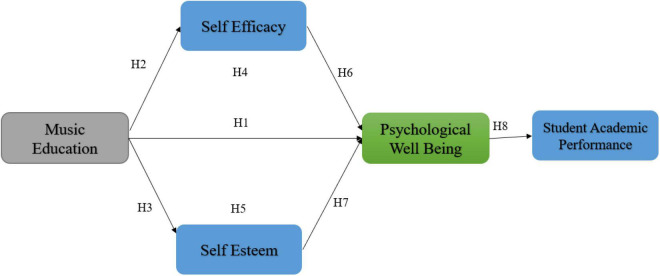
Conceptual model.

This study is based on the following conceptual framework (see Figure 1).

## Methodology

The population of this research study are students studying in schools, colleges, and universities in China. This study is quantitative and empirical in nature. A survey questionnaire was designed to collect data from respondents. A total 350 questionnaires were distributed out of which only 319 questionnaires were received back so that the response rate of this study is 91%. Out of 319 respondents, 160 were boys/men and 159 respondents were girls/women. Similarly, 85 respondents were below the age of 10–15 years, 95 respondents between 16 and 20, and 130 respondents between 20 and 30 as depicted in Table 1. Similarly, the qualifications of 190 respondents were undergraduate, while 129 respondents were graduates. The convenience sampling technique was used for data collection because it is the easiest way to collect data from the respondents (Nawaz et al., 2019; Dar et al., 2022). Therefore, due to limited time, this study employs a convenience sampling technique. A total of 25 items were utilized from the literature to design a questionnaire for this study. Music education was measured by a five-item scale adopted from Dönmez et al. (2019). The psychological wellbeing was measured by a five-item scale adopted from Diener et al. (2009). Furthermore, self-esteem was measured by a four-item scale adopted from Rosenberg (1989). Similarly, self-efficacy was assessed by a five-item scale from Schwarzer and Jerusalem (1995). Students’ performance was measured by a six-item scale adopted from Rashid and Zaman (2018). This study is quantitative and cross-sectional. The partial least square (PLS) method was used for data analysis. A statistical software Smart-PLS used for data analysis.

**TABLE 1:** Demographic details.

Valid		Frequency	Percent	Valid percent	Cumulative percent
	Female	159	49.8	49.8	49.8
	Male	160	50.2	50.2	100.0
	Total	319	100.0	100.0	
	10–15	85	26	26	26.6
	16–20	95	30	30	56.0
	20–30	139	44	44	100.0
	Total	319	59.5	100.0	
	Undergraduate	159	40.5	59.5	59.5
	Graduate	160	100.0	40.5	100.0
	Total	319		100.0	

*N = 319.*

## Data Analysis and Results

This research study’s data was conducted using PLS methodology and statistical software Smart-PLS was used for data analysis. Data analysis consists of two stages, i.e., measurement analysis and structural analysis. In the first step, the measurement model was used to investigate the reliability, convergence, and validity of the construct. Finally, structural equation modeling (SEM) was used for testing of hypotheses.

### Measurement Model

**FIGURE 2:**
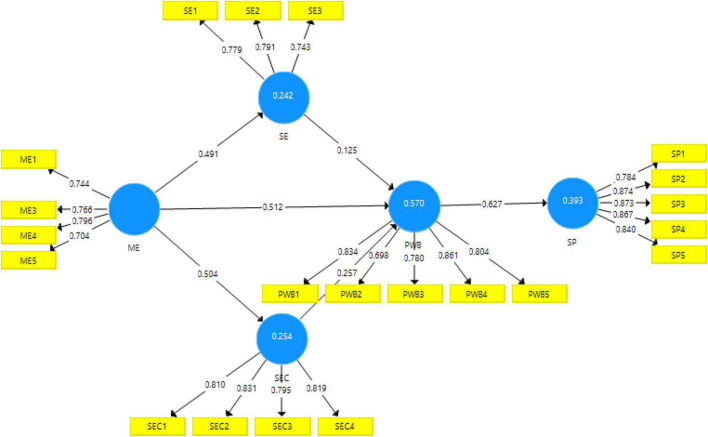
Output of measurement model algorithm.

The measurement model of the constructs was examined in the first step to determine the reliability, convergence, and discriminant validity of latent constructs. In this study, latent variables are assessed by the observed indicators (items) and are reflective in nature. In the reflective model, indicators are affected by the latent variable in the other words arrows are toward the indicators from their latent variables as can be seen in Figure 2 measurement model in which arrows point from latent variables toward indicators. In the First step, indicator loading was measured for each construct. It has been proposed that if the value of outer loadings is greater than 0.70, it is acceptable, which means that construct represents the 70% of items that construct (Hair et al., 2019; Avotra et al., 2021b). However, some studies suggest that a value greater than 0.50 is also an acceptable reliability (Hulland, 1999). In this study as indicated in Table 2, all outer loading is greater than 0.50 which shows reliability, except for ME2, SE4, SEC5, SP6 which were removed from the model to generate better results. Cronbach’s alpha, composite reliability and rho_A test were used for investigating the internal consistency of the construct. According to Hair et al. (2019), Cronbach’s alpha’s value should be higher than 0.7 to establish the internal consistency of constructs. Table 2 illustrates that Cronbach’s alpha of each latent construct is higher than 0.70 which shows higher internal consistency of scale. The second method that determines the internal reliability and consistency of a scale is composite reliability. All Composite Reliability (CR) values greater than 0.7 indicate Internal Consistency (Hair et al., 2019; Yingfei et al., 2021). Table 2 highlights that the composite reliability of each latent construct is above 0.70 thus internal consistency is established. Third method for measuring reliability is rho_A. The value of rho_A is greater than 0.70 is acceptable for determining reliability (Hair et al., 2019; Xiaolong et al., 2021). Table 2 reflects that all value of rho_A is greater than 0.70 thus internal consistency is established.

**TABLE 2:** Reliability and convergent validity.

Construct	Items	Outer loading	Alpha	rho_A	CR	AVE
Psychological wellbeing			0.855	0.861	0.897	0.636
	PWB1	0.834				
	PWB2	0.698				
	PWB3	0.780				
	PWB4	0.861				
	PWB5	0.804				
Self-esteem			0.831	0.834	0.887	0.662
	SE1	0.779				
	SE2	0.791				
	SE3	0.743				
Self-efficacy			0.902	0.903	0.928	0.720
	SEC1	0.810				
	SEC2	0.831				
	SEC3	0.795				
	SEC4	0.819				
Student performance						
	SP1	0.784				
	SP2	0.874				
	SP3	0.873				
	SP4	0.867				
	SP5	0.840				
Music education			0.746	0.751	0.84	0.568
	ME1	0.744				
	ME3	0.766				
	ME4	0.796				
	ME5	0.704				

*CA, Cronbach’s alpha; CR, composite reliability; AVE, average variance extracted. ME, Music education; PWB, Psychological wellbeing; SE, Self-esteem; SEC, Self-efficacy; SP, Student’s performance.*

Convergent validity refers to all items of the constructs that are closely related to each other (Hair et al., 2012). The average variance extracted (AVE) is used for determining convergent validity for all constructs. The threshold value of AVE is equal or greater than 0.5 to establish the convergent validity of constructs (Hair et al., 2019). In this study, AVE for all the variables is above 0.50. Thus, convergent validity has been established.

### Discriminant Validity

Discriminant validity refers to each construct being empirically distinct from other constructs. Discriminant validity discusses the differentiation of each latent variable from other variables. There are three methods to determine discriminant validity in Smart-PLS: Fornel and lacker criteria, Cross loadings, and Heterotrait-Monotraite. According to Fornell and Larcker (1981), to establish the discriminant validity of constructs the square root of AVE of each variable in the research model must be higher than the correlation of the same variable with others. Table 3 indicates that the square root of AVE of each construct is greater than the diagonal values below. Hence, discriminant validity is established.

**TABLE 3:** Fornell and Larker.

	ME	PWB	SE	SEC	SP
**ME**	**0.753**				
**PWB**	0.703	**0.797**			
**SE**	0.491	0.501	**0.771**		
**SEC**	0.504	0.575	0.483	**0.814**	
**SP**	0.519	0.627	0.45	0.548	**0.848**

*ME, Music education; PWB, Psychological wellbeing; SE, Self-esteem; SEC, Self-efficacy; SP, Students performance. Bold values shows the significance between the variables.*

Another method to determine discriminant validity is cross-loading. After the Fornell and Larker method, cross-loading is an authentic method for determining discriminant validity. Criteria for this technique is that the values of each item with its own construct are higher as compared to other constructs. If the value of item is highly correlated to their own construct compared to other constructs, then discriminant validity is established in Table 4.

**TABLE 4:** Cross-loading.

	ME	PWB	SE	SEC	SP
ME1	***0.744***	0.606	0.293	0.349	0.437
ME3	***0.766***	0.553	0.448	0.443	0.489
ME4	***0.796***	0.52	0.361	0.397	0.344
ME5	***0.704***	0.427	0.373	0.317	0.266
PWB1	0.623	***0.834***	0.436	0.507	0.507
PWB2	0.506	***0.698***	0.407	0.369	0.459
PWB3	0.469	***0.78***	0.323	0.432	0.481
PWB4	0.571	***0.861***	0.422	0.507	0.516
PWB5	0.615	***0.804***	0.400	0.564	0.532
SE1	0.425	0.395	***0.779***	0.357	0.313
SE2	0.356	0.351	***0.791***	0.423	0.345
SE3	0.35	0.409	***0.743***	0.341	0.386
SEC1	0.364	0.433	0.355	***0.810***	0.469
SEC2	0.374	0.421	0.32	***0.831***	0.451
SEC3	0.451	0.484	0.379	***0.795***	0.402
SEC4	0.437	0.519	0.498	***0.819***	0.464
SP1	0.445	0.528	0.352	0.431	***0.784***
SP2	0.452	0.553	0.383	0.447	***0.874***
SP3	0.458	0.546	0.437	0.481	***0.873***
SP4	0.401	0.528	0.372	0.507	***0.867***
SP5	0.442	0.502	0.363	0.459	***0.840***

*ME, Music education; PWB, Psychological wellbeing; SE, Self-esteem; SEC, Self-efficacy; SP, Students performance. Bold and italic values shows the significance of variables.*

Heterotrait-Monotrait ratio of correlations (HTMT) is another method for evaluating discriminant validity of the data in SEM proposed by Dijkstra and Henseler (2015). If the values of HTMT are high, then the discriminate value problems arise. The threshold value of HTMT is 0.9 proposed by Dijkstra and Henseler (2015) which means two variables are correlated but not more than 0.9. All values of HTMT in Table 5 have a value less than 0.9, which reflects that the discriminant validity of constructs have been established.

**TABLE 5:** HTMT ratio.

	ME	PWB	SE	SEC	SP
ME	0.753				
PWB	0.703	0.797			
SE	0.491	0.501	0.771		
SEC	0.504	0.575	0.483	0.814	
SP	0.519	0.627	0.45	0.548	0.848

*ME, Music education; PWB, Psychological wellbeing; SE, Self-esteem; SEC, Self-efficacy; SP, Students performance.*

### Collinearity Statistics (VIF)

Variance inflation factor values are used to investigate the collinearity Issues and common method biasness of structural model. According to Hair et al. (2019) VIF is an indicator which is used to measure whether all indicator variables are correlated to each other or not and to avoid all issues regarding the significance and its value must be less than three (Hair et al., 2019). In our results, all values are less than three as shown in Table 6. Hence, we conclude that there are no collinearity issues between the variables in the proposed structural model.

**TABLE 6:** Collinearity statistics (VIF).

	VIF
ME1	1.383
ME3	1.419
ME4	1.649
ME5	1.438
PWB1	2.128
PWB2	1.454
PWB3	1.855
PWB4	2.522
PWB5	1.869
SE1	1.283
SE2	1.406
SE3	1.24
SEC1	2.084
SEC2	2.217
SEC3	1.614
SEC4	1.707
SP1	1.933
SP2	2.76
SP3	2.772
SP4	2.803
SP5	2.527

*ME, Music education; PWB, Psychological wellbeing; SE, Self-esteem; SEC, Self-efficacy; SP, Students performance.*

Model fitting parametric was tested before going for the structural assessment model. Model fits parametric includes SRMR (Standardized Root Mean Square Residual) and NFI (Normed fit indices). SRMR refers to “the difference between the observed correlation and the model implied correlation matrix whereby values are less than 0.08” (Hu and Bentler, 1998). In this study value of SRMR is greater than 0.8 which meets the required criteria. The second model fit parametric is Normed fit indices (NFI), greater than 0.90. Value of normed fit indices is 0.901 which is acceptable. The structure equation model (SEM) provides the means that shows the hypothesized path by supporting the theoretical model. Basically, SEM model comprises with the hypothesized relationship between the independent and dependent variables in the projected research model. The structural model predicts that how well the theoretical model envisages the hypothesized pathways. For the current study, SEM Model is assessed with the coefficient of determination (*R*^2^), Coefficient of determination (*R*^2)^ measures the variation in the dependent variable due to independent variables. *R*^2^-value of 0.75, 0.50, and 0.25 are considered substantial, moderate (Hair et al., 2019). Table 7 shows the value of 0.570 for PWB which is strong. This shows that all independent variables have 57% variance in psychological wellbeing whereas value of 0.242 for self-esteem shows 24% variance in self-esteem due to all independent variables. Value of 0.242 for self-efficacy shows 25% variance in self-efficacy due to all independent variable. Moreover, value of 0.394 for student performance shows 39% variance in student performance due to all independent capacity is established.

**TABLE 7:** Coefficient of determination (*R*^2^).

	*R* ^2^	SD	*T*-value	*P*-values
PWB	0.570	0.050	11.448	0.000
SE	0.242	0.049	4.930	0.000
SEC	0.254	0.044	5.816	0.000
SP	0.394	0.046	8.645	0.000

*PWB, Psychological wellbeing; SE, Self-esteem; SEC, Self-efficacy; SP, Students performance.*

Hypothesis1 proposed that there is a relationship between music education and psychological wellbeing. Result indicates that Music education has a significant and positive effect on psychological wellbeing (β = 0.512, *t* = 9.010, *p* = 0.000). As the value of *p* < 0.05, therefore this hypothesis is accepted. Hypothesis 2 proposed that there is a relationship between music education and self-esteem. Our result indicates that the ME has a significant and positive effect on self-esteem (β = 0.491, *t* = 9.873, *p* = 0.000). As the value of *p* < 0.05, therefore this hypothesis is accepted. Hypothesis 3 proposed that there is a relationship between music education and self-efficacy. Results indicate that music education has significant and positive effect on self-efficacy (β = 0.504, *t* = 11.492, *p* = 0.000). As the value of *p* < 0.05, therefore this hypothesis is accepted. Hypothesis 4 proposed that there is a relationship between self-esteem and psychological wellbeing. Results indicate that the SE has a significant and positive effect on psychological wellbeing (β = 0.125, *t* = 2.386, *p* = 0.017). As the value of *p* < 0.05, therefore this hypothesis is accepted. Hypothesis 5 proposed that there is a relationship between self-efficacy and psychological wellbeing. The result indicates that the SEC has a significant and positive effect on PWB (β = 0.257, *t* = 5.306, *p* = 0.000). As the value of *p* < 0.05, therefore this hypothesis is accepted. Hypothesis 8 proposed that PWB has significant positive impacts on SP. Results indicate that the PWB has a significant and positive effect on Students’ performance (β = 0.627, *t* = 17.247, *p* = 0.000). As the value of *p* < 0.05, therefore this hypothesis is accepted (Table 8).

**TABLE 8:** Hypotheses constructs.

Hypotheses	Relationship	Beta coefficient	*SD*	*T*-value	*P*-values	2.50%	97.50%	Decision
H1	ME - > PWB	0.512	0.057	9.010	0.000	0.386	0.616	Supported
H2	ME - > SE	0.491	0.05	9.873	0.000	0.391	0.583	Supported
H3	ME - > SEC	0.504	0.044	11.492	0.000	0.405	0.586	Supported
H4	SE - > PWB	0.125	0.052	2.386	0.017	0.022	0.233	Supported
H5	SEC - > PWB	0.257	0.048	5.306	0.000	0.163	0.355	Supported
H8	PWB - > SP	0.627	0.036	17.247	0.000	0.560	0.697	Supported

*ME, Music education; PWB, Psychological wellbeing; SE, Self-esteem; SEC, Self-efficacy; SP, Student’s performance.*

This study proposed the mediating role of self-esteem and self-efficacy between the relationship of Music education and Students’ psychological wellbeing. Our research applied Preacher and Hayes (2008) method for mediation analysis which is the most powerful and rigorous method for mediation analysis. Hypothesis 6 proposed that SEF mediates the relationship between ME and PWB The result shows that SEF positively mediates the relationship between ME and PWB (β = 0.061, *t* = 2.337, *p* = **0.020**) as shown in Table 8. As the value of *p* < 0.05, therefore this hypothesis is accepted. In Hypothesis 7, we proposed that SEF mediates the relationship between ME and PWB. Results illustrate that SEF positively mediates the relationship between ME and PWB (β = 0.129, *t* = 4.851, *p* = 0.000) as shown in Table 7. As the value of *p* < 0.05, therefore this hypothesis is accepted (Table 9 and Figure 2).

**TABLE 9:** Mediation analysis.

Hypotheses	Relationship	Beta coefficient	*SD*	*T*-value	*P*-values	2.50%	97.50%	Decision
H6	ME - > SE - > PWB	0.061	0.026	2.337	0.020	0.011	0.111	Supported
H7	ME - > SEC - > PWB	0.129	0.027	4.851	0.000	0.079	0.182	Supported

*ME, Music education; PWB, Psychological wellbeing; SE, Self-esteem; SEC, Self-efficacy; SP, Students performance.*

## Discussion

This research revolved around certain objectives based on set goals to check the impact of music education on the wellbeing of the students psychologically and their academic performance during the most depressing moments of Pandemic. This study yielded some interesting results supporting the model of this research. The model is based on the remedial purpose of combating the depressing effects of COVID-19. Our first hypothesis was about checking the relationship between music education and Students’ psychological wellbeing, which has been previously tested and proved that music has a specific remedial effect on individuals of every walk of life. Our hypothesis was also accepted suggesting a strong relationship between psychological wellbeing and music education. These results are in accordance with many past studies such as Croom (2014) and Demirbatır (2015). The possibility of this kind of result is due to the soothing nature of music itself. In the period of online classes, socialization was limited to digital sources only, so music education was also possible over the internet. It helped in fighting the distress caused by COVID-19 during these times. The second and third hypotheses were about the impact and relationship of music education with self-esteem and the self-efficacy of the students.

As discussed earlier in the review of literature section, many researchers including Faulkner et al. (2010), Barbre (2013), and Rickard et al. (2013) indicated that music education was positively related to Students’ self-esteem and self-efficacy, which leads to academic achievement and the psychological wellbeing of the students. Our results were in accordance with these researchers’ results due to the importance of music in developing self-esteem and a sense of self-ability in students during these pandemic times. The fourth hypothesis was about the relationship of self-esteem with the Students’ psychological wellbeing. This hypothesis was also supported in the sense of this relationship. This happens because self-esteem plays an integral role in developing a strong sense of self-confidence in psychology, which leads to the Students’ psychological wellbeing. These results are also in accordance with many past researchers reporting similar results from different perspectives (Barbre, 2013; Sowislo and Orth, 2013).

The relationship of Students’ self-efficacy with psychological wellbeing also resulted in the same outcomes as self-efficacy is also associated with the Students’ self-esteem and it leads to Students’ psychological wellbeing during his pandemic. These results are in agreement with (Bandura et al., 2003; Siddiqui, 2015). The hypotheses about the mediating roles of self-esteem and self-efficacy were also accepted between the relationships of music education and the psychological wellbeing of the students. As it is well established that both self-esteem and self-efficacy had and have a strong relationship in positively improving the psychological wellbeing of the students, so the mediating role between music learning and the wellbeing of students psychologically was understood to play a significant role due to the nature of them. These results were in accordance with many past researchers who analyzed the mediating roles of both self-esteem and the self-efficacy of the students (Molero et al., 2018; Sabouripour et al., 2021).

The last hypothesis was about the relationship of the psychological wellbeing of the students with their academic performance. It is a well-understood concept that if a student is psychologically active, efficient, and has good mental wellbeing then it would result in excellent performance, leading to distinction in academic performance. Psychological wellbeing is directly related to elevated grades or academic achievement. The last hypothesis yielded the same results and confirmed the results of many researchers in the past such as Bhullar et al. (2014) and Roslan et al. (2017). The results obtained through this study are a great indicator of teaching music at institutes for developing better psychological wellbeing of the students to achieve improved academic performance from students.

## Theoretical Contributions

This study contributes to the body of literature in a significant way. First, only very limited previous studies have examined the cause and effects of the student psychological and mental health, especially during crises. Recently, coronavirus has affected every part of society, so like others, students were also affected badly so there was a dire need to explore this topic and present solid solutions to mitigate the anxiety, stress, and burnout among undergraduate and graduate students. This study reveals that music education plays a vital role in improving Students’ psychological wellbeing, which ultimately leads to improving the Students’ performance. This study conceptualizes self-determination theory, self-esteem theory, and social cognitive theory to investigate the conceptualized path. The finding of this study reveals that music education develops self-efficacy and self-esteem in the students which assists them in improving their psychological wellbeing.

## Practical Contributions

The results of this study can assist policymakers and practitioners in realizing the importance of music education in the curriculum of undergraduate and graduate students, which has long-lasting impacts on their psychological wellbeing and practical life, so there is a dire need to include music education in the curriculum. In addition to this, results show that self-efficacy and self-esteem also play a vital role in improving Students’ psychological wellbeing, so educational institutions should make their efforts to develop a sense of self-esteem and self-efficacy through their curriculum, training, and other means. Proper training should be provided to teachers of education institutes to help the students mitigate the issues of anxiety, stress, and depression, which are the main obstacles for students to become successful in their academic and professional lives.

## Limitation and Future Direction

Besides the contribution of this study, there are some limitations of this study that could be mitigated in future studies to attain better results. First, the sampling technique adopted for this study is the convenience sampling technique, so future studies should adopt any other rigorous sampling technique. The sample size of this study was very small due to the lockdown enforced by the government to mitigate the COVID-19 pandemic. This study was cross-sectional in nature so future studies should adopt time-series studies to get better results. Future researchers should explore other factors that may assist in improving psychological wellbeing as well as other impacts and consequences of music education. Future studies should explore other mediators and moderators in the relationship between music education and psychological wellbeing.

## Conclusion

Recent scientific studies reveal the effects of pandemic and its associated illness which influence people’s mental health and psychological wellbeing. Music education and its practices are recognized as a vital tool in reducing the level of stress and anxiety among the students which has a significant impact on their personality grooming and academic performance. The main purpose of this study is to estimate the impact of music education on the psychological wellbeing and academic performance of students. This study also investigates the mediating role of self-esteem and self-efficacy between this relationship. This study adopts the theoretical lens of social cognitive, self-esteem theory, and self-determination theory to explain the proposed model of this study. This study is quantitative in nature and data of this study was collected from 319 students who are studying in undergraduate and graduate institutions by using self-administered questionaries. Convenience sampling was used in the data collection. PLS-SEM technique was used for data analysis. The results show that music education has a significant impact on Students’ psychological wellbeing and academic performance. Self-esteem and self-efficacy mediate the relationship between music education and psychological wellbeing. Results of this research study contribute to the body of literature on music education and psychological wellbeing and explore new avenues for future studies. Policy-makers and practitioners should promote music education in educational institutes to improve Students’ psychological wellbeing and academic performance.

## Data Availability Statement

The original contributions presented in the study are included in the article/supplementary material, further inquiries can be directed to the corresponding author/s.

## Ethics Statement

The studies involving human participants were reviewed and approved by the Xihua University, China. The patients/participants provided their written informed consent to participate in this study. The study was conducted in accordance with the Declaration of Helsinki.

## Author Contributions

JS contributed in all tasks of writing of draft and data collection etc.

## Conflict of Interest

The author declares that the research was conducted in the absence of any commercial or financial relationships that could be construed as a potential conflict of interest.

## Publisher’s Note

All claims expressed in this article are solely those of the authors and do not necessarily represent those of their affiliated organizations, or those of the publisher, the editors and the reviewers. Any product that may be evaluated in this article, or claim that may be made by its manufacturer, is not guaranteed or endorsed by the publisher.
